# Validation of model predicting furcation involvement in newly crowned teeth—A 5‐year retrospective follow‐up

**DOI:** 10.1002/jper.70072

**Published:** 2026-02-07

**Authors:** Khushboo Kalani, Giuseppe Troiano, Asfandyar Sheikh, Era Kakar, Hom‐Lay Wang, Muhammad H. A. Saleh

**Affiliations:** ^1^ Department of Periodontics and Oral Medicine University of Michigan School of Dentistry Ann Arbor Michigan USA; ^2^ Department of Clinical and Experimental Medicine University of Foggia Foggia Italy

**Keywords:** dental prosthesis, furcation defects, periodontal attachment loss, periodontitis, validation study

## Abstract

**Background:**

This study aimed to perform a prediction model validation for furcation involvement (FI) risk in molars receiving a new fixed prosthesis (FP) using a unique cohort assessed at three time points.

**Methods:**

Following the Oral Health Statistical (OHStat) reporting guidelines, this cohort study examined 181 patients (203 molars) from 2018–2023. Teeth without FI were followed longitudinally post‐crown placement at 1– (T1), 3‐ (T2), and 5‐years (T3). A logistic regression model was built in order to predict FI and the related performance was assessed through metrics like AUC, sensitivity, specificity, and calibration.

**Results:**

FI was observed in 4.43% of teeth at 1 year, increasing to 21.67% at 3 years and 28.57% at 5 years. Univariate analysis revealed significant predictors at 3–5 years: a history of periodontitis was associated with higher FI risk at 5 years (RR = 3.56, *p* = 0.024), with advanced stages also increasing risk—stage III: RR = 2.59 at 3 years and RR = 3.32 at 5 years; stage IV: RR = 3.76 at 3 years and RR = 3.75 at 5 years. Short root trunks significantly increased FI risk across all intervals (1 year: RR = 3.96; 3 years: RR = 6.08; 5 years: RR = 4.75). Medium trunks did not differ significantly from long trunks. The predictive model performed best at 3 years (AUC = 0.81, sensitivity = 0.79, specificity = 0.87) and remained robust at 5 years (AUC = 0.76, sensitivity = 0.69, specificity = 0.90).

**Conclusion:**

The predictive model demonstrated high accuracy with a substantial ability to identify FI cases over time. Clinicians should consider such an assessment before crown or bridge restoration, with particular caution in patients with periodontitis.

## Plain language summary

This study performed a retrospective validation for a predictive model for furcation involvement (FI), which showed high accuracy with a considerable ability to identify FI cases over time, which was the strongest at 3 years (AUC = 0.81).

## 1 INTRODUCTION

Periodontitis progression is a multifaceted, multifactorial quandary.^1^, ^2^, ^3^ FI is a well‐known risk predictor contributing to lower prognostic scores in various prognostication tools^4^ and is strongly associated with tooth loss.^5^ Thus, it is crucial to consider the risk of FI in teeth while planning treatment for patients with periodontitis.^6^

The complexities that fixed dental prostheses add to teeth with FI necessitate thorough examination and discussion within the literature.^7^ Crowned molars pose a high risk for furcation involvement due to several factors: improper margin placement, overextension of restorations, short root trunk, altered access for hygiene, and potential biological width violation. These factors can lead to increased susceptibility to periodontal progression. Hence, assessing FI risk specifically in crowned molars aids in targeted preventive planning and prosthetic design. Wang et al.’s landmark study examined the relationship between restorations and furcation involvement by focusing solely on clinical aspects such as crown presence, root exposure, furcation involvement (FI), tooth mobility (MO), and attachment level (AL) through periodontal charting. However, the study did not incorporate radiographic bone‐level assessment, staging of periodontitis, or key anatomical and systemic risk factors, limiting its ability to quantify FI risk comprehensively.^8^

We recently included systemic factors (like diabetes and smoking) and all the tooth level factors that might contribute to FI, such as distance from crown margin to furcation entrance, root trunk length, probing depth, supracrestal soft tissue height, supracrestal tissue attachment^9^, interproximal bone distance, anatomical site of furcation, presence of prosthesis, periodontal stage and grade in a prediction model to determine the risk of FI.^10^ The model incorporated advanced cone‐beam computed tomography (CBCT) imaging, which enabled precise assessments of root anatomy, supracrestal soft tissue height, and FI risk assessment. The suggested model included significantly associated factors, such as periodontitis stage and root trunk length, to determine the probability of FI for crowned teeth. It exhibited a high sensitivity of 92.9%, a moderate specificity of 66.7%, and an overall area under the curve of 0.86, indicating the model's reliable predictability for FI in teeth with prosthetic crowns.^10^

Risk prediction models support clinical decision‐making by identifying high‐risk patients and informing them about preventive strategies. However, validation is critical for such models to be applicable in clinical settings. Initial validation, often called internal validation, is typically performed using the same data set on which the model was developed. While helpful, this approach may not reflect the model's performance in other settings or time points. Therefore, temporal or external validation must confirm the model's reproducibility across varying patient demographics and clinical practice environments.^11^, ^12^ When deployed in real‐world practice, a model will inevitably be tested on new data from different cohorts, even if from the same population base,^13^ opening doors for later external validation.^4^

Thus, this study aimed to temporally validate our previously developed model using a distinct cohort treated across a different timeframe and evaluated with 2D imaging, allowing for real‐world applicability testing. This validation uses a distinct cohort from a distinct time point assessed at three follow‐up time points.

## 2 METHODS

The study was conducted per the principles outlined in the 1975 Declaration of Helsinki and received approval from the Institutional Review Board for Human Studies (IRBMED: HUM00228878) at the University of Michigan School of Dentistry. The manuscript is prepared following OHStat (Oral Health Statistical reporting guidelines).^14^ The validation of this model also drew upon the Transparent Reporting of a Multivariable Prediction Model for Individual Prognosis or Diagnosis (TRIPOD) statement as a point of reference.

### 2.1 Study population

Patients who underwent dental crown placement from 2018 to 2023 at the University of Michigan School of Dentistry, Ann Arbor, Michigan, United States, were included in this retrospective cohort study. The data were obtained from the electronic health records (EHR) of 181 patients, including 203 molar teeth with single‐unit fixed prostheses in the analysis (Figure S1 in the online *Journal of Periodontology*). The study was partially funded using the same university's Periodontics and Oral Medicine department research fund. No written consent from the participants was required since data from electronic health records were de‐identified before use.

### 2.2 Eligibility criteria

The following inclusion criteria were applied:

1. Patient with at least one dental crown on any of the first or second molars (Maxillary or mandibular).
2. Molar tooth without FI at the time of crown placement.
3. A baseline T0 with crowns and subsequent three follow‐up periods, T1(1 year following the date of the crown placement) and T2 (3 years following the date of the crown placement) and T3(5 years following the date of the crown placement).
4. Patients with periodontal charts within 1 month (±) of radiographs at each time point.

Patients were excluded from the study if they met any of the following criteria:

1. Fixed prosthesis (FP) on only the third molar,
2. Veneer or partial crown,
3. Tooth extracted due to reasons other than periodontitis before the follow‐up time point recorded,
4. Teeth treated with endodontic therapy,
5. Incomplete or missing clinical records, periodontal charts, and radiographs at any follow‐up point (± 1 month).

### 2.3 Allocation of FI diagnosis

Furcation involvement was verified from periodontal charts at the time of crown placement (± 1 month) in the first and second molars, and horizontal furcation depth was evaluated using a Nabers probe at furcation entrances. Upper molars were assessed at mid‐buccal, mesiopalatal, and distopalatal entrances; lower molars were examined from mid‐buccal and mid‐lingual sites. The classification of FI presence and severity followed the criteria that Hamp et al. outlined^15^:

Degree I: horizontal attachment loss < 3 mm of the total width of the furcation area.
Degree II: horizontal attachment loss > 3 mm but not encompassing the total width of the furcation area.
Degree III: “through and through” destruction of the periodontal tissue in the furcation area.

### 2.4 Data collection and patient classification

Three examiners (AS, EK, and KK) screened and assessed Axium electronic health records (EHR) (Exan, Henry Schein, Melville, New York, USA) of patients who received dental crowns at the University of Michigan School of Dentistry and were deemed eligible per the abovementioned criteria. All patients included in this retrospective cohort were on periodontal maintenance, as evidenced by comprehensive periodontal charting at each follow‐up. Data collection occurred between August 2023 and October 2023, and individuals meeting the specified inclusion criteria were included in the study. Patients' demographic data were collected, and patient‐level factors such as sex, age at the time of crown placement, history of smoking, and diabetic status were included from the EHR. Using patient periodontal charts and radiographs, the periodontal diagnosis was made according to the 2017 World Workshop on the Classification of Periodontal and Peri‐Implant Diseases and Conditions.^16^ Staging was determined on a full mouth, with the most severely affected tooth guiding classification. Importantly, molars without baseline furcation involvement were eligible for inclusion, even if a stage III/IV diagnosis was based on non‐molar teeth (e.g., anterior or premolars with ≥5 mm interproximal attachment loss or tooth loss attributable to periodontitis). Baseline periodontal charting confirmed the absence of furcation involvement in the included molars.

All relevant patient, tooth, and site‐level information was collected from the EHR. The validation cohort assessed root trunk length using 2D periapical and/or bitewing radiographs, reflecting the standard diagnostic modality used in routine clinical practice. In contrast, the development study used CBCT imaging. Like the previous study, the associated root trunk lengths were categorized as short, medium, or long based on two criteria. First, it was gauged according to the methodology Hou, Chen, Tsai, and Weisgold outlined.^17^ This system evaluates root trunks by comparing their vertical height to the total root length, categorizing them as Type A, B, and C, corresponding to root trunks in the cervical, middle, and apical thirds of the total root length.^17^, ^18^ Second, it follows the Ochsenbein classification. For maxillary molars, the classifications are short (3 mm or less), medium (4 mm), and long (5 mm or more). In contrast, mandibular molars are categorized as short (2 mm or less), medium (3 mm), and long (4 mm or more).^19^ The primary analytic level was the tooth and using periodontal charts and bitewing and/or periapical radiographs, tooth level factors, including probing depth, root trunk length, and FI (predicted outcome) were assessed and recorded at three separate time points: baseline (without FI), T1 (1‐year follow‐up), T2 (3 years follow‐up), and T3 (5 years follow‐up). The assessors were not blinded to the predictor values.

### 2.5 Prediction model

The first study developed and internally validated a predictive model for FI in crowned teeth.^10^ Independent factors and covariates were analyzed using multiple binary logistic regression, and model optimization was performed through backward elimination. Internal validation was performed using 20‐fold cross‐validation. The dataset was split into 95% training and 5% test sets for each fold. Model performance was averaged across folds, including AUC, accuracy, sensitivity, specificity, and calibration metrics (Table S1 in the online *Journal of Periodontology*).

Periodontitis stage was the strongest predictor, with significantly increased risk in advanced stages (stage III: OR = 22.3, *p* = 0.003; stage IV: OR = 6.65, *p* = 0.095). Buccal root trunk length also influenced FI probability, with medium trunks reducing risk (OR = 0.16, *p* = 0.024). The final model demonstrated high sensitivity (92.9%) and moderate specificity (66.7%), with an AUC of 0.86 (*p* < 0.001), confirming its predictive performance.

The model estimates FI probability (*p*), allowing different cutoff points to balance sensitivity and specificity. Receiver Operating Characteristic^20^ analysis identified multiple cutoff points for ‘*p*’ (0.42, 0.50, 0.62, 0.66, and 0.77), permitting adjustments based on research aims.^21^ A cutoff of p = 0.50 provides high sensitivity (92.9%) and moderate specificity (66.7%), while *p* = 0.62 and *p* = 0.66 improve specificity (70.0% and 76.7%, respectively) at the cost of sensitivity. The final prediction rule was *p* ≤ 0.5 for non‐FI and *p* > 0.5 for FI. Employing this rule, the model demonstrated an overall correct classification rate of 85%, with positive predictive value (PPV) and negative predictive value (NPV) values of 86.7% and 80%. Since our model predicts a binary outcome, discrimination was assessed using the area under the curve (AUC), corresponding to the c‐statistic (Table S2 in the online *Journal of Periodontology*).

To facilitate clinical application, an Excel‐based tool was developed (Figure S2 in the online *Journal of Periodontology*), enabling clinicians to input patient and tooth data for FI prediction based on selected cut‐off points.

### 2.6 Statistical analysis

#### 2.6.1 Sample size

The validation set sample size was calculated using Calibration‐in‐the‐Large and AUC metrics.^22^ The expected event proportion was 15%, targeting a standard error (SE (ln(O/E) of 0.175, resulting in a minimum sample size of 185 participants. An expected C‐statistic of 0.80 for the AUC aimed for a confidence interval width of 0.175 (SE(C) = 0.0446). An iterative approach with the nonparametric SE(C) formula determined the required sample size to be 194 participants.

#### 2.6.2 Analytical approach

Descriptive statistics were performed for both predictors and outcomes analyzed; categorical variables expressed frequencies and percentages, while continuous variables presented means with standard deviations. The study involved a complete case analysis. Records were excluded if periodontal charts or radiographs were missing at any follow‐up point (± 1 month). This conservative approach aimed to minimize data inconsistency. No imputation was performed. Univariate analyses were conducted, and each predictor was tested separately using a multilevel mixed‐effects logistic regression framework to account for the hierarchical structure of the data (subject, tooth, furcation). The association between predictors and outcomes (FI at 1, 3, and 5 years)‐predicted probabilities from a prior model‐ history of periodontitis, stage of periodontitis, and root trunk length^10^ calculated the risk of FI at the tooth level during follow‐up. Model performance evaluation included discrimination (AUC metrics), PPV, NPV, specificity, sensitivity, and calibration (slope, in large, BRIER score)^23^, ^24^, ^25^ after applying bootstrap validation with 1000 replications to the cohort. A *p* value under 0.05 indicated statistical significance.

## 3 RESULTS

### 3.1 Patients’ cohort and clinical variables

A cohort consisting of 203 crowned molars in 181 patients (96 females and 85 males) with a mean age of 58.99 ± 9.72 and a mean follow‐up of 0.89 ± 0.42 at T1, 3.06 ± 0.36 at T2, and 5.08 ± 0.37 at T3 was included in this study (Table 1). Among the included patients, 15.47% (28/181) were smokers, 83.43% (151/181) had a history of periodontitis, 23.20% (42/181) were stage I, 51.38% (93/181) were stage II, 17.12% (31/181) were stage III, and 2.76% (5/181) were stage IV, while 40.88% (74/181) were grade A, 52.48% (95/181) were grade B, and 1.10% (2/181) were grade C. 16.6% (30/181) of the patients were diabetic. At the tooth level, 40.88% (83/203) were maxillary molars and 58.62% (119/203) were mandibular molars. The root trunk lengths were categorized as short in 14.29% (29/203) of the teeth, medium in 73.89% (150/203), and long in 11.82% (24/203). The mean maximum probing depth (PDMax) observed at baseline was 3.69 ± 0.87 mm at T1, 3.64 ± 0.94 mm at T2, and 3.62 ± 1.01 mm at T3 (Figure S3 in the online *Journal of Periodontology*).

**TABLE 1:** Demographic characteristics.

Variables	Michigan cohort
**No. of patients**	181
**No. of teeth**	203
Maxillary molars	83/203 (40.88%)
Mandibular molars	119/203 (58.62%)
**Sex**	
Men	85 (46.96%)
Women	96 (53.04%)
**Age (years)**	58.99 ± 9.72
**Smoking**	
Yes	28 (15.47%)
No	153 (84.53%)
**History of periodontitis**	
Yes	151 (83.43%)
No	30 (16.57%)
**Stage**	
I	42 (23.20%)
II	93 (51.38%)
III	31 (17.12%)
IV	5 (2.76%)
**Grade**	
A	74 (40.88%)
B	95 (52.48%)
C	2 (1.10%)
**Diabetes**	
Yes	30 (16.57%)
No	151 (83.43%)
**Root Trunk (Ochsenbein, 1986** ^19^ **)**	
Short	29 (14.29%)
Medium	150 (73.89%)
Long	24 (11.82%)
**PDMax**	
T1	3.69 ± 0.87
T2	3.64 ± 0.94
T3	3.62 ± 1.01
**Mean follow‐ups**	
T1	0.89 ± 0.42
T2	3.06 ± 0.36
T3	5.08 ± 0.37

### 3.2 Timing‐based FI

FI was observed in only 4.43% (9/203) of the teeth at 1‐year follow‐up (T1), indicating a relatively low initial occurrence. However, as the follow‐ups progressed, there was a significant increase in the incidence of FI. At 3 years of follow‐up (T2), 21.67% (44/203) of the teeth exhibited FI (*p* = < 0.001), while at 5 years of follow‐up (T3), the incidence significantly increased further, with 28.57% (58/203) of the teeth showing FI (*p* = < 0.001) compared with (T1) (Table 2).

**TABLE 2:** Predictions and outcomes evaluated in this study at 1‐, 3‐, and 5‐year follow‐ups.

Variables	Teeth (*n* = 203)	*p*‐value
**Furcation predicted**		
No	149 (73.40%)	
Yes	54 (26.60%)	
**Furcation involvement at T1 (1 year)**		**T1 vs. T2**
No	194 (95.57%)	*p* < 0.001
Yes	9 (4.43%)	
**Furcation involvement at T2 (3 years)**		**T2 vs. T3**
No	159 (78.32%)	*p* = 0.137
Yes	44 (21.67%)	
**Furcation involvement at T3 (5 years)**		**T1 vs. T3**
No	145 (71.43%)	*p* < 0.001
Yes	58 (28.57%)	

### 3.3 Analysis of FI

The univariate analysis (Table 3) revealed several significant predictors for FI at 3‐to 5‐year follow‐up intervals. Notably, a history of periodontitis was significantly associated with a higher risk of FI at the 5‐year follow‐up (RR = 3.56 [1.18‐10.73], *p* = 0.024). Stage of periodontitis was also a significant predictor. At 3‐year follow‐up, stage III and IV periodontitis showed significantly higher risk of FI than stages I‐II, with a RR = 2.59 [1.02–6.57] with a *p*‐value of 0.025 and RR = 3.76 [2.23–6.32] with a *p*‐value of 0.001, respectively. This association was further accentuated at the 5‐year follow‐up, where the RR changed to 3.32 [1.78–6.22] with *p*‐value of 0.001 for stage III periodontitis. Similar results were obtained for stage IV, showing a high RR of 3.75 [1.88–7.47] with *p* = 0.001 at the 5‐year follow‐up. Additionally, root trunk length significantly reduced the risk of FI. Root trunk length was significantly associated with furcation involvement at all follow‐up intervals. Compared with long root trunks, teeth with short trunks had a markedly higher risk of FI: at 1 year (RR = 3.96; 95% CI 1.08–14.58; *p* = 0.038), at 3 years (RR = 6.08; 95% CI 1.89–19.49; *p* = 0.002), and at 5 years (RR = 4.75; 95% CI 1.60–14.13; *p* = 0.005). In contrast, medium root trunks were not significantly different from long trunks at any time point (1 year: RR = 0.46 [95% CI 0.11–1.89], *p* = 0.281; 3 years: RR = 0.91 [95% CI 0.23–3.65], *p* = 0.899; 5 years: RR = 1.14 [95% CI 0.31–4.27], *p* = 0.843.

**TABLE 3:** Univariate binomial generalized linear regression models with log link for the outcome furcation involvement at 1‐, 3‐, and 5‐ years of follow‐up after applying bootstrap internal validation with 1,000 iterations.

	Univariate Analysis								
Furcation Involvement	1‐year			3‐years			5‐years		
	RR	[95% CI]	*p*‐value	RR	[95% CI]	*p*‐value	RR	[95% CI]	*p*‐value
**Age**	0.99	[0.93 – 1.07]	0.959	1.02	[0.98 – 1.04]	0.263	1.01	[0.98 – 1.03]	0.751
**History of periodontitis**									
No	1.			1.			1.		
Yes	1.55	[0.47–5.12]	0.470	2.65	[0.86–8.18]	0.089	3.56	[1.18–10.73]	0.024^*^
**Sex**									
Men	1.			1.			1.		
Women	2.90	[0.77–10.83]	0.133	1.32	[0.74–2.34]	0.348	1.02	[0.64–1.62]	0.933
**Smoking**									
No	1.			1.			1.		
Yes	0.67	[0.20–2.22]	0.508	1.57	[0.84–2.95]	0.159	1.39	[0.82–2.37]	0.223
**Diabetes**									
No	1.			1.			1.		
Yes	1.53	[0.41–5.69]	0.528	0.53	[0.17–1.63]	0.271	0.73	[0.32–1.67]	0.462
**Stage**									
I‐II	1.			1.			1.		
III	1.89	[0.37–4.23]	0.327	2.59	[1.02–6.57]	0.025^*^	3.32	[1.78–6.22]	0.001^*^
IV (172 patients)	2.47	[0.89–4.16]	0.145	**3.76**	[2.23–6.32]	0.001^*^	**3.75**	[1.88–7.47]	0.001^*^
**Root trunk length**									
Long	1.			1.			1.		
Short	3.96	[1.08–14.58]	0.038^*^	6.08	[1.89–19.49]	0.002^*^	4.75	[1.60–14.13]	0.005^*^
Medium	0.46	[0.11–1.89]	0.281	0.91	[0.23–3.65]	0.899	1.14	[0.31–4.27]	0.843
**PDmax**	0.67	[0.32–1.39]	0.287	1.04	[0.79–1.36]	0.770	1.17	[0.90–1.52]	0.229

Abbreviation: RR: Risk ratio.

* *p* value = 0.054

### 3.4 Performance of the prediction model

Model performance metrics (Table 4) in terms of discrimination capability. At the 1‐year follow‐up, the model performed similarly at all the cut‐off points analyzed. At the 0.66 cut‐off, it achieved a sensitivity of 0.55 and a specificity of 0.87, with a PPV of 0.16 and a high NPV of 0.97. The AUC was 0.77, resulting in a moderate overall discriminative power (Figure 1).

**TABLE 4:** Sensitivity, specificity, positive predictive value (PPV), negative predictive value (NPV), and area under the curve (AUC) for predicting furcation involvement at 1‐year, 3‐year, and 5‐year follow‐ups.

Cut‐off	Sensitivity	Specificity	PPV	NPV	AUC	Time point
0.42	0.78	0.75	0.13	0.98	0.77	1‐year follow‐up
0.5	0.78	0.75	0.13	0.98		
0.62	0.66	0.83	0.15	0.98		
0.66	0.55	0.87	0.16	0.97		
0.77	0.55	0.77	0.16	0.97		
0.42	0.79	0.87	0.64	0.94	0.81	3‐year follow‐up
0.5	0.79	0.87	0.64	0.94		
0.62	0.50	0.89	0.54	0.86		
0.66	0.45	0.93	0.63	0.86		
0.77	0.43	0.92	0.61	0.85		
0.42	0.70	0.90	0.73	0.88	0.76	5‐year follow‐up
0.5	0.69	0.90	0.73	0.88		
0.62	0.47	0.92	0.69	0.81		
0.66	0.40	0.94	0.72	0.80		
0.77	0.38	0.94	0.91	0.79		

*Note*: The metrics are reported for different cut‐off points (0.42, 0.50, 0.62, 0.66, 0.77), indicating the model's performance correctly identifying positive and negative cases over time.

Abbreviations: AUC, area under the curve; NPV, negative predictive value; PPV, positive predictive value.

**FIGURE 1:**
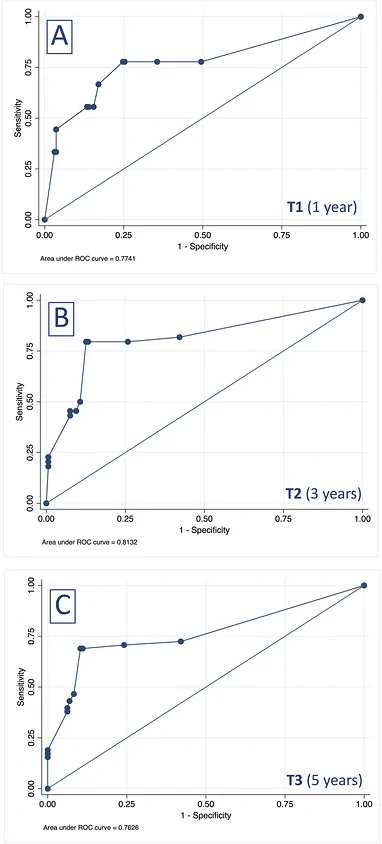
ROC curve illustrating the model's performance in predicting furcation involvement at A) T1 (1 year), B) T2 (3 years), and C) T3 (5 years). The *x*‐axis represents the false‐positive rate (1—specificity), and the *y*‐axis represents the true‐positive rate (sensitivity). The blue curve shows the model's discriminative ability at various cut‐offs, with an AUC of 0.77, 0.81, and 0.76, respectively.

At the 3‐year follow‐up, the model performed optimally at a cut‐off point 0.42. This cut‐off yielded a sensitivity of 0.79 and a specificity of 0.87. The PPV was 0.64, while the NPV was 0.94. The AUC for this follow‐up was 0.81, demonstrating good discriminative power (Figure 1).

At the 5‐year follow‐up, the model's best performance was observed at a cut‐off point 0.50. This cut‐off provided a sensitivity of 0.69 and a specificity of 0.90, with a high NPV of 0.88 and a PPV of 0.73. The AUC was 0.76, indicating solid overall predictive accuracy (Figure 1).

Regarding calibration, the model showed varying performance across the follow‐up periods. At the 1‐year follow‐up, the Calibration‐in‐the‐large (CITL) was −2.21, with a Calibration Slope of −0.016, indicating a significant miscalibration. The Brier Score was 0.322, reflecting moderate accuracy. By the 3‐year follow‐up, calibration improved slightly, with a CITL of −1.61 and a Calibration Slope of −0.378. The Brier Score was 0.332. At the 5‐year follow‐up, the Calibration Slope improved to 0.374, with a CITL of −2.52 and a Brier Score of 0.316, suggesting persistent significant miscalibration and suboptimal predictive accuracy over time.

## 4 DISCUSSION

In this validation (temporal) study, assessing the accuracy of a FI predictive model of crowned teeth observed a progressive increase in furcation involvement over time, rising from 4.43% at the 1‐year follow‐up to 28.57% at 5 years post‐crown placement. Significant predictors for the development of FI were again a history of advanced periodontitis—stages III and IV—and shorter root trunk lengths. The previously developed model demonstrated good discriminative ability with an area under the curve of up to 0.81 at the 3‐year follow‐up. Our results demonstrate the necessity of evaluating periodontitis history and root trunk length as essential factors before prescribing full‐coverage crown restorations for molar teeth.

Accurate evaluation of risk demonstrates the importance of preventive measures, leading to the adoption of less invasive treatment plans. Early and precise risk assessment enables clinicians to implement targeted strategies to halt disease progression.^26^ Ultimately, emphasizing prevention enhances patient oral health and lowers long‐term health care costs.^27^ This longitudinal retrospective cohort study aimed to assess the validity of a furcation risk prediction model specifically for crowned molars and its association with bone levels. The findings reinforce that FI is a notable risk predictor for periodontally compromised teeth fitted with FP. Over the study's observation period, a progressive increase in FI was observed, particularly at the 3‐year and 5‐year follow‐up points. Although FI Grade I may not significantly alter treatment decisions in many cases, it was included in this study as an early indicator of interradicular breakdown. Its identification supports risk stratification, especially in maintenance patients, where early intervention may delay progression to more advanced stages. Nevertheless, future models may benefit from focusing on more clinically significant endpoints such as FI Grade II or III, which directly impact prognosis and treatment planning.^28^, ^29^ From a clinical perspective, incorporating FI risk prediction into treatment planning can guide modifications in prosthetic design, crown margin placement, or preventive maintenance protocols. While FI alone may not contraindicate crown placement, as it still may be needed for reasons such as structural protection or restorative necessity, and in such cases the prosthetic plan can be tailored (e.g., onlays, supragingival margins) to minimize periodontal risk. Instead, our methodological focus was identifying incident FI cases after crown placement in previously unaffected molars, thereby enabling risk prediction based solely on progression rather than pre‐existing involvement. Overall, the present study's findings are consistent with those of earlier studies.^5^, ^6^, ^10^, ^30^ These studies indicate that advanced stages of periodontitis (stages III and IV) have significantly increased odds of developing FI over time. Hirschfeld and Wasserman's retrospective analysis spanning 22 years demonstrated that among patients receiving periodontal treatment, a significant majority were diagnosed with advanced disease and indicated that teeth with FI were associated with higher tooth loss rates compared with molars without FI unless classified as “extremely downhill” in the study.^31^ Another investigation over 10 years showed that 79.5% of tooth extractions involved multi‐rooted teeth, while only 20.5% pertained to single‐rooted teeth.^32^ However, a retrospective study provided optimistic findings regarding the survival rates of multirooted teeth affected by FI, which varied between 42.9% and 100% depending on the treatment type, including guided tissue regeneration and root resection.^33^

Regarding tooth‐specific factors, the odds associated with the present study's root trunk length align with Hou and Tsai's earlier findings. Specifically, teeth with short root trunks exhibited the highest susceptibility to FI, as even modest attachment loss rapidly exposes the furcation entrance. In contrast, medium and long root trunks were associated with reduced odds of FI at all follow‐up intervals, reflecting a relative protective effect. This highlights the importance of root anatomy in the stability of multi‐rooted teeth, particularly regarding crown placement.^18^, ^34^

The predictive model showed moderate to high discriminative ability, with AUC values of 0.76–0.81, indicating good predictive accuracy for FI.^35^ No significant differences emerged among cut‐off points; lowering the cut‐off did not significantly affect sensitivity, specificity, PPV, or NPV. However, increasing the cut‐off resulted in higher specificity and NPV but lower sensitivity. This trend shows that higher cut‐off points make the model more conservative, enhancing its capacity to identify cases without FI (increased specificity and NPV) while reducing the detection of all cases resulting in FI (decreased sensitivity). This sensitivity‐specificity trade‐off emphasizes the model's adaptability for minimizing false positives or maximizing true positives.^36^, ^37^ Additionally, calibration analysis revealed slight miscalibration in the early 1‐year follow‐up, with improvements over time. The findings align with Troiano et al.,^38^ who documented performance metrics over ten years, and support Steyerberg's,^25^ [Ch. 10] assertion that extended follow‐up enhances calibration and discrimination.^25^, ^38^, ^39^

This validation study emphasizes the predictive validity of the FI risk model and its potential application in clinical decision‐making processes. Notably, the model is not intended to serve as a contraindication for crown placement nor as the sole determinant for extraction procedures.^40^ Instead, it is an objective assessment tool that enables clinicians to stratify patient risk and incorporate preventive strategies into comprehensive treatment planning.^41^, ^42^ Ultimately, this model serves as a translational bridge linking predictive research outcomes to everyday clinical decision‐making within prosthodontic and periodontal practice. Beyond its predictive accuracy, it provides clinicians with a structured framework to better understand the biological mechanisms and rate of furcation progression, especially in cases where direct longitudinal data are lacking. For patients identified as high‐risk, clinicians may consider adopting more conservative prosthetic approaches, such as using supragingival margins when appropriate, alongside implementing more rigorous supportive periodontal care intervals or multidisciplinary consultations to evaluate alternative restorative options when prognosis remains uncertain. This approach facilitates shared decision‐making, ensuring patients are adequately informed of the biological risks and the potential long‐term implications of various treatment options.^43^

Among the few studies that investigate the correlation and prediction of FI with prosthesis, it has certain limitations. Firstly, the retrospective design introduces selection bias, and the lack of external validation from other populations, since the data are from a single institution, limits generalizability. An external cohort could enhance this, but using the same institution restricts the applicability of the findings in broader clinical contexts. Furthermore, the 5‐year follow‐up threshold may contribute to selection bias. Secondly, FI serves as a surrogate endpoint for tooth loss, which may not hold significant weight in treatment planning, especially if patients are still determining whether to proceed with crowning or extraction. For instance, if a tooth can be restored with an onlay, FI status alone might not influence the treatment decision. Thirdly, although essential clinical variables are integrated into the model, it does not consider genetic predispositions or systemic conditions, limiting its applicability in diverse patient populations. In addition, due to the limited sample size in our validation cohort, performing a full multivariate analysis was not feasible. A further limitation is the lack of precise data on glycemic control in diabetic patients in our cohort, although all treated cases were assumed to be systemically controlled.

Fourth, a standardized endodontic follow‐up was not performed; however, endodontically treated molars were excluded, and EHR review (symptoms/sensibility/radiographs) did not identify new endodontic diagnoses among included teeth during the observation window.

Fifth, the periodontitis staging was conducted retrospectively using patient records, unlike the prospective clinical assessments used in the development study. This methodological difference may hinder the accurate identification of certain complexity factors.

Sixth, baseline differences between the development and validation cohorts were not directly analyzed, limiting the understanding of model performance heterogeneity. Future prospective validations should include demographic and clinical comparisons to assess external generalizability. Seventh, while the initial model development utilized CBCT measurements for high precision, our validation study depended on 2D imaging techniques. These techniques better represent real‐world practice, where CBCT isn't commonly employed. Although this difference in measurement methods may have led to inconsistencies, it emphasizes the model's effectiveness and functionality in realistic clinical environments, rather than its external generalizability across diverse settings. Finally, a major limitation is the model's significant miscalibration across follow‐up periods, reflected by highly negative CITL values, slopes far from 1, and high Brier scores. These results indicate overestimation and instability of predictions, underscoring the need for recalibration and cautious interpretation of predictive performance.

The model's temporal validity also calls for external validation in a clinical setting involving more significant, more diverse patient populations, as Schwendicke et al. also recommended.^44^ When externally validated in prospective studies, such models could aid in treatment planning by identifying high‐risk teeth, thus enabling clinicians to focus on preventing tooth loss. Additionally, future research should aim to integrate additional factors such as restorative material type, endodontic outcomes, and occlusal analysis for comprehensibility.

## 5 CONCLUSION

In conclusion, this study validates (temporal) a furcation risk prediction model with high predictive accuracy over time, offering clinicians a valuable tool for assessing FI in molars with crowns. However, given the miscalibration across the follow‐ups, results should be interpreted cautiously. Future studies must prospectively refine the model and confirm its external validity in diverse settings to ensure that it is effective in clinical practice, prioritizing the prevention of periodontal bone loss in the furcal area over time through risk assessment before crown placement.

## AUTHOR CONTRIBUTIONS

**Muhammad H. A. Saleh**: Conceptualization; supervision. **Khushboo Kalani, Asfandyar Sheikh, and Era Kakar**: Data curation; investigation. **Giuseppe Troiano**: Formal analysis; statistical analysis. **Khushboo Kalani and Giuseppe Troiano**: Writing—original draft preparation. **Hom‐Lay Wang and Muhammad H. A. Saleh**: Writing—review; editing & supervision.

## CONFLICT OF INTEREST STATEMENT

The authors do not have any financial interests, either directly or indirectly, in the products or information identified in the paper.

## Supporting information



Supporting Information

## Data Availability

The data that support the findings of this study are available from the corresponding author upon reasonable request.
