# Supplementary material for: Validation of model predicting furcation involvement in newly crowned teeth—A 5‐year retrospective follow‐up
Source: J Periodontol. 2026 Feb 7;97(10):2033–43. doi: 10.1002/jper.70072 (PMC13743499; doi:10.1002/jper.70072)
Supplement: Supplementary file 1 [file JPER-97-2033-s001.docx]

## **Supplemental Figures & Tables**

Supplemental Figure 1: Flowchart showing the patient selection process.

**Figure S2:** : [Excel sheet snap including predictions for different cutoff points from this model](https://static-content.springer.com/esm/art%3A10.1007%2Fs00784-024-05787-z/MediaObjects/784_2024_5787_MOESM4_ESM.png) with user instructions.

**^[1]^https://sites.google.com/umich.edu/fi-prediction-model/home**

Figure S3: Furcation involvement (FI) prevalence and probing depth (PD Max) over time. *(Panel A) FI prevalence stratified by maxillary and mandibular molars at T1, T2, and T3 follow-ups, presented with standard error of the mean error (SEM). (Panel B) Average PD Max (mm) stratified by maxillary and mandibular molars across T1, T2, and T3, with SEM.*

Table S1: Sensitivity, specificity, PPV, and NPV at different cutoff values.

|  | **Cutoff p=0.42** | **Cutoff p=0.50** | **Cutoff p=0.62** | **Cutoff p=0.66** | **Cutoff p=0.77** |
| --- | --- | --- | --- | --- | --- |
| **Sensitivity** | 92.9% | 92.9% | 90.0% | 84.3% | 81.4% |
| **Specificity** | 66.7% | 66.7% | 70.0% | 76.7% | 76.7% |
| **PPV** | 86.7% | 86.7% | 87.5% | 89.4% | 89.1% |
| **NPV** | 80.0% | 80.0% | 75.0% | 67.6% | 63.9% |
| **% corrected cases** | 85.0% | 85.0% | 84.0% | 82.0% | 80.0% |
| **AUC** | 0.86  (0.77-0.94); **p<0.001***** | | | | |

Table S2: Internal vs. external validation comparison

| **Metric** | **Internal Validation** | **External Validation (1-Year)** | **External Validation (3-Year)** | **External Validation (5-Year)** |
| --- | --- | --- | --- | --- |
| **AUC** | 0.86 | 0.77 | 0.81 | 0.76 |
| **Sensitivity (p=0.50)** | 92.9% | 78% | 79% | 69% |
| **Specificity (p=0.50)** | 66.7% | 75% | 87% | 90% |
| **PPV (p=0.50)** | 86.7% | 13% | 64% | 73% |
| **NPV (p=0.50)** | 80% | 98% | 94% | 88% |
| **Correct Classification Rate** | 85% | Moderate | High | High |

1. https://sites.google.com/umich.edu/fi-prediction-model/home
